# Nutrients, Cognitive Function, and Brain Aging: What We Have Learned from Dogs

**DOI:** 10.3390/medsci9040072

**Published:** 2021-11-18

**Authors:** Yuanlong Pan

**Affiliations:** Nestlé Purina Research, 2RS, 1 Checkerboard Square, St. Louis, MO 63164, USA; yuanlong.pan@rd.nestle.com; Tel.: +1-314-982-3707

**Keywords:** aging, atrophy, brain, cognitive function, cortical, ketone bodies, learning, medium-chain triglycerides, memory, neurogenesis

## Abstract

Due to a difference in genetics, environmental factors, and nutrition, just like in people, dogs age at different rates. Brain aging in people and dogs share similar morphological changes including irreversible cortical atrophy, cerebral amyloid angiopathy, and ventricular enlargement. Due to severe and irreversible brain atrophy, some aging dogs develop cognitive dysfunction syndrome (CDS), which is equivalent to dementia or Alzheimer’s disease (AD) in people. The risk factors and causes of CDS in dogs have not been fully investigated, but age, gender, oxidative stress, and deficiency of sex hormones appears to be associated with increased risk of accelerated brain aging and CDS in dogs. Both AD and CDS are incurable diseases at this moment, therefore more efforts should be focused on preventing or reducing brain atrophy and minimizing the risk of AD in people and CDS in dogs. Since brain atrophy leads to irreversible cognitive decline and dementia, an optimal nutritional solution should be able to not only enhance cognitive function during aging but also reduce irreversible brain atrophy. Up to now, only one nutritional intervention has demonstrated both cognition-enhancing benefits and atrophy-reducing benefits.

## 1. Introduction

Brain aging leads to similar morphological and functional changes in both people and dogs [1]. The main changes in the brains of dogs include significant and irreversible loss of brain cells, significant global cortical atrophy, cerebral amyloid angiopathy (CAA) with compromised cerebral blood flow, and ventricular enlargement [2,3,4,5,6]. Aged dogs developed diffuse plaques and hyperphosphorylated tau in the brains [7,8]. However, neurofibrillary tangles were not observed in the brains of aged dogs [7]. The ability to learn simple tasks such as simple associative learning is not affected in healthy senior dogs, however, senior dogs have decreased ability to learn prefrontal-dependent tasks [1]. Just like in humans, senior dogs can be classified into successful agers, mild cognitive impairment, and severe cognitive impairment called cognitive dysfunction syndrome (CDS) [9,10]. With increased longevity in dogs, higher incidence of CDS has been reported in senior dogs [11,12,13]. Clinical symptoms in dogs with CDS include disorientation, reduced social interaction, increased anxiety, disrupted sleep/wake cycle, change in activity, and loss of house training [13,14,15,16]. Since the aging-induced neurodegenerative process is gradual and irreversible, more efforts should be made to develop solutions that are able to slow down brain aging and brain atrophy [17,18] and an optimal nutritional solution should be able to both enhance cognitive function and reduce the irreversible brain atrophy. Many risk factors are associated with brain aging and increased risk of Alzheimer’s disease (AD) [17,18]. We proposed that managing multiple known risk factors should lead to more effective nutritional solutions for healthy brain aging than the most common approach of addressing a single risk factor. By targeting these risk factors, we have developed two nutritional solutions, medium chain triglycerides (MCT) [19] and the brain protection blend (BPB) [17,18] to enhance cognitive function and slow aging-induced cognitive decline in dogs and/or cats. In addition, our recent data indicates that the BPB can reduce brain atrophy in cats. A combination of the MCT and BPB was also able to alleviate the clinical symptoms of dogs with CDS [20]. Since brain aging in dogs and humans shows many similarities, our nutritional solutions for dogs may help to develop comparable solutions to promote healthy brain aging, reduce the risk of AD, and manage clinical symptoms of AD in people.

## 2. Aging-Related Morphological Changes in the Brains of Dogs

Brain aging in dogs results in several morphological changes similar to those observed in aging brains of humans [1,4,5,21,22,23]. The main morphological changes in aged dogs include significant and irreversible loss of brain cells, significant global cortical atrophy, cerebral amyloid angiopathy (CAA) with compromised cerebral blood flow, and ventricular enlargement [2,3,4,5,6,24]. Su et al. [5] examined the changes in brain morphometrics of 47 dogs (8–11 years old) over three years and observed both ventricular volume enlargement and significant lesions, especially in the frontal cortex and caudate nucleus of the brain in old dogs. Various brain regions appear to undergo aging-related atrophy at different rates and the frontal lobes are particularly sensitive to the aging-related neurodegenerative process [24]. For instance, significant atrophy occurred in the frontal lobe of old dogs (8–11.4 years of age) compared with young dogs (six months to 3.9 years of age), significant hippocampal atrophy was observed in senior dogs (11.5 years and old) compared with young dogs, but no age-associated atrophy was observed in the occipital lobe in the dogs [24]. In addition, significant frontal lobe atrophy correlated with impaired executive function and increased beta-amyloid deposition in the frontal cortex [24]. White matter myelin loss was also observed in the brains of senior dogs [25], which is consistent with the age-dependent loss of cortical myelin in the brains of normal people [26].

Existing evidence suggests that cerebrovascular lesions are involved in brain aging and the pathogenesis of dementia in both people and dogs [6,27,28,29]. Cerebral amyloid angiopathy (CAA) is one of the histological hallmarks in the brains of AD patients [30]. CAA may lead to chronic cerebral hypoperfusion, impair microcirculation, and lead to microbleeds and infarcts associated with cognitive decline [31]. In a study by Uchida et al. [29], CAA was reported in all older dogs, with cerebral hemorrhage detected in 66% of the older dogs and massive hemorrhage in 22%.

Brain atrophy and ventricular enlargement observed in senior dogs are mostly due to irreversible loss of brain cells, and partly caused by decreased neurogenesis. Significant neuron loss was observed in the brains of old dogs [32,33]. A number of factors have contributed to the significant neuron loss including apoptosis, cerebral vascular lesion, increased oxidative stress, and chronic inflammation [34,35,36,37,38]. Senior dogs have significant reduction in neurogenesis compared with young adult dogs [39,40]. Reduced neurogenesis was due to decreased progenitor cell proliferation and reduced survival of new neurons and was irreversible [39].

Senile amyloid plaques and neurofibrillary tangles are the neuropathological hallmarks of AD [41]. Age-related accumulation of diffuse plaques similar to the early stage of beta-amyloid peptide deposits in humans and hyperphosphorylated tau were observed in aged dogs [7,8]. However, neurofibrillary tangles are not observed in aged dogs [3,7]. Head et al. [42] reported that Abeta-42 accumulation was present in the brain earlier than Abeta-40 and correlated with an increased amyloid load. In the CSF samples, Abeta-42 declined with age and Abeta-40 remained stable in dogs [42].

Since the aging-induced neurodegenerative process is gradual and irreversible, it is imperative to start interventions earlier to reduce neuron loss and promote healthy brain aging.

## 3. Aging-Related Metabolic Changes in the Brains

Brains are highly metabolically active organs, and account for about 25% of daily whole-body glucose consumption [43]. Sustaining higher neuronal activity depends on more ATP supply from increased cerebral glucose metabolism [44]. Therefore, maintaining adequate energy supply is critical for normal brain functions. Reduced cerebral glucose metabolism has been reported in healthy old people [45], old rodents [46], old dogs [47], and old monkeys [48]. Cerebral glucose metabolism was significantly reduced even in middle-aged rats and dogs compared with young adult animals [46,47]. Since no significant brain atrophy has been reported in middle-aged dogs and rats, the reported reduction in cerebral glucose metabolism in middle-aged dogs and rats is not due to the loss of brain cells [46,47]. Cerebral glucose metabolism is further compromised in subjects with AD compared with age-matched healthy old subjects [49,50]. Existing evidence indicates that insulin, insulin-like growth factor-1 (IGF-1) and their receptors are involved in regulating metabolism, neuronal growth, learning and memory functions, and protecting neurons against cell death [51,52]. Moreover, insulin and insulin receptors decreased with aging in the brain, which suggests that dysfunction in insulin signaling pathways may be, at least partly, responsible for the reduced glucose metabolism associated with aging and AD [53,54]. This hypothesis was further supported by the link between type 2 diabetes mellitus and high risk of AD [55,56,57]. Since cerebral glucose metabolism correlates strongly with cognitive function [44], reduced cerebral glucose metabolism may partly contribute to the decline in brain functions associated with aging and AD [50,54,58]. Impaired cerebral glucose metabolism can result in compromised brain function. It was proposed that more efforts should be focused on improving the cerebral energy state to reduce the risk of AD [54]. One of the options is to increase ketone body supply as an alternative energy source for the brain with ketogenic diets or medium chain triglycerides (MCT) [59,60,61].

## 4. Aging-Related Cognitive and Behavioral Changes in Dogs

Milgram [62] reported that cognitive function decreased significantly in middle-aged dogs compared with young dogs. Mild impairments of spatial learning and memory can be detected as early as six years in dogs [63]. The ability to learn simple tasks such as simple associative learning is not affected in healthy senior dogs, however, senior dogs have decreased ability to learn prefrontal-dependent tasks [1]. Senior dogs showed deficits in learning, memory, executive function, and attention [21,64,65,66]. Based on cognitive performance, senior dogs can be classified into successful agers, mild cognitive impairment, and cognitive dysfunction syndrome (CDS) [9,10]. Cortical atrophy leads to aging-related changes in brain functions and there is a strong correlation between cortical atrophy and decline in cognitive functions in old dogs [24]. There is a significant correlation between the extent of Aβ deposition and declined in cognitive function in dogs [1,7]. Aβ load was significantly correlated with several cognitive tasks including discrimination learning, reversal learning, and spatial learning in dogs [7]. More interestingly, higher Aβ load was significantly correlated with errors of learning knowledge-based tasks, but no correlation was observed between Aβ load and errors of learning skill-based tasks in dogs [7]. Reduced frontal lobe volume significantly correlated with increased Aβ load in the frontal cortex and with impaired executive function including inhibitory control and complex working memory in old dogs [24]. The number of phosphorylated tau protein (p-tau)-positive cells in the brains were much higher in dogs with CDS than in the normal aged dogs [8]. In addition, the levels of p-tau increased with the ages of dogs with CDS and were significantly associated with the Aβ deposition in dogs with CDS [8]. The number of newly formed neurons in the hippocampus significantly correlated with cognitive functions in dogs [39]. These data suggest that neurogenesis may be involved in the process of learning and memory formation and reduced neurogenesis may contribute to the declined cognitive function [39]. Since cerebral glucose metabolism correlates strongly with cognitive function [44], reduced cerebral glucose metabolism may contribute to the decline in brain functions also associated with aging and AD [50,58].

Just like in humans, some senior dogs eventually develop cognitive impairment or cognitive dysfunction syndrome (CDS). Clinical symptoms in dogs with CDS include disorientation, reduced social interaction, increased anxiety, disrupted sleep/wake cycle, change in activity, and loss of house training [12,13,14,15,16]. About 27.5% of old dogs from 11 to 12 years of age developed mild to severe cognitive impairment and the incidence was 67% for dogs between 15 to 16 years of age [13]. Changes in social interactions, disrupted sleep–wake cycle, and general activity were the most common signs, followed by disorientation and house soiling in dogs with CDS [67]. Another study reported that changes in social interaction (37.7%) and loss of house training (37.7%) were the most common signs, followed by disrupted sleep/wake cycle (20.2%) and disorientation (16.4%) in dogs with CDS [11]. CDS adversely affects the quality of life of both dogs and their owners. Cognitive impairment and CDS are the consequences of severe and irreversible brain atrophy induced by aging in senior dogs.

## 5. Risk Factors for Brain Aging and Cognitive Dysfunction Syndrome

Many risk factors have been associated with brain aging and AD, but risk factors associated with brain aging and CDS in dogs have not been fully explored. Age has been reported to be a risk factor for AD in people [68] and CDS in dogs [13,69]. In dogs, the prevalence does not differ between breed size, even though large breed dogs have a shorter lifespan than small breed dogs [13,69].

Gender appears to be a significant risk factor for AD in people and the incidence of AD in females is almost twice of that in male subjects [70]. Similarly, the incidence of CDS in female dogs was more than twice that in male dogs [11]. Menopause is associated with increased risk of AD in people [71,72]. Hart [73] conducted a study to evaluate the effects of gonadectomy on CDS development in dogs. The results indicated that intact male dogs were significantly less likely to progress from mild impairment to CDS than neutered dogs, and both neutered male dogs and spayed female dogs showed similar rates of progression from mild impairment to CDS. Another study showed that neutered dogs were almost twice more affected than intact dogs [11]. Both estrogen and androgen have been shown to have neuroprotective effects [72,74]. These data suggest that sex hormone deficiency may be a risk factor for brain aging and dementia in people and dogs.

Several risk factors have been associated with brain aging and increased risk of AD in people. Some of the risk factors can be eliminated or mitigated by nutritional interventions including DHA deficiency [75], high homocysteine [76], inadequate status of vitamin B6, vitamin B12, and folic acid [77,78], oxidative stress [79,80], and chronic low grade inflammation [81,82]. In addition, high blood pressure [83], cerebral vascular lesion [84], diabetes [85], brain injuries [85], and obesity [86] have also been associated with increased risk of brain aging and AD in humans. Both cardiovascular disease and AD share similar risk factors including hypertension, high homocysteine, oxidative stress, chronic inflammation, diabetes, and obesity, which suggests that vascular factors may play an important role in the pathogenesis of AD. Cerebrovascular lesions lead to chronic brain hypoperfusion, which results in loss of brain cells, setting the stage for age-related cognitive decline and AD development [27,28]. Cerebral ischemia may lead to sporadic AD by causing neuronal loss, beta-amyloid accumulation, and tau pathology [87,88,89]. Increased oxidative damage in the brains of old dogs suggests that oxidative stress may be a contributing factor for brain aging and CDS in dogs [36,90]. Since brain aging in people and dogs share many similarities [1], it is reasonable to speculate that the above-mentioned risk factors observed in people may also apply to dogs.

Risk of cardiovascular diseases has been significantly reduced by mitigating or eliminating known risk factors [91]. A similar approach should be used to retard the irreversible process of brain atrophy, promote successful brain aging, and reduce the risk of dementia. We proposed that brain aging and atrophy may be slowed down by eliminating or reducing known risk factors associated with brain aging and AD [92], and our studies in dogs and cats indicate that this approach is able to delay age-related cognitive decline in dogs and cats [17,18].

## 6. Nutritional Solutions for Healthy Brain Aging and CDS Management

Many factors including genetics, environment, and nutrition may determine the different rates of brain aging observed in people and dogs. A dietary regimen including higher intake of fruits, vegetables, fish, nuts and legumes, and lower intake of meats, high fat dairy, and sweets was associated with lower odds of cognitive deficits or reduced risk of AD [93]. We hypothesized that compensating for the early cerebral metabolic change and mitigating known risk factors associated with brain aging and dementia may be effective in promoting successful brain aging, reducing brain atrophy, and minimizing the risk of dementia [17,18,92].

### 6.1. Ketone Bodies as an Alternative Energy Source for the Brain

Normal cerebral glucose metabolism is essential for cognitive function [44], unfortunately, cerebral glucose metabolism is impaired with age and in subjects with cognitive impairment [45,49,50]. Reduced cerebral glucose metabolism may partly contribute to the decline in brain functions associated with aging and cognitive impairment in AD subjects [50,54,58]. Since reduced cerebral glucose metabolism occurs under the condition of normal blood glucose and appears to be caused by dysfunction of the insulin signaling pathways [53,54], further increasing blood glucose does not help to improve cerebral energy supply. However, increasing ketone body supply as an alternative energy source for the brain appears to be able to enhance cognitive function in people with cognitive impairment [59,60,61].

Ketone bodies (acetoacetate, acetone, and beta-hydroxybutyrate) are mainly produced in the liver from long chain fatty acids during glucose shortage. Acetoacetate and beta-hydroxybutyrate can be interconverted and utilized by many tissues. In addition to glucose, the brains can also utilize ketone bodies as energy when blood ketone bodies are available. A human study showed that ketone bodies accounted for 60% of the brain’s energy requirement during starvation [94]. Cerebral metabolism of ketone bodies depends on the levels of ketone bodies in the blood and the number of monocarboxylic acid transporters 1 (MCT1) of the blood–brain barrier (BBB). MCT1 transport ketone bodies across the BBB into the brain [95]. MCT1 are also present on glia, while monocarboxylic acid transporters 2 (MCT2) are exclusively found on neurons [96].

When glucose supply from diets is adequate, carnitine palmitoyl transferase is inhibited by malonyl-CoA to support the storage of energy surplus as triglycerides [97]. Therefore, the traditional ketogenic diets usually contain high fat, low protein, and extremely low carbohydrates [98]. However, the ketogenic diets may not serve as a long-term solution for old people or animals because of the side effects [99].

Medium-chain triglycerides (MCT) are composed of C6, C8, C10, and C12 saturated fatty acids. MCT have several unique properties compared with long-chain triglycerides. First, MCT do not require bile salts for digestion. Second, resulting medium-chain fatty acids (MCFA) are absorbed more efficiently than long chain fatty acids (LCFA) and transported in the portal blood directly to the liver, while LCFA need to be incorporated into chylomicrons and transported through the lymphatic system. Third, MCFA diffuse into the mitochondria independently of carnitine palmitoyl transferase and are rapidly converted into ketone bodies in the liver [100]. Therefore, dietary MCT can increase blood ketone body levels regardless of the levels of dietary carbohydrates, fat, and proteins. Increased blood levels of ketone bodies can increase brain metabolism of ketone bodies as an alternative energy source to compensate for the deficits in cerebral glucose metabolism. This increases the energy supply to the brain and helps to maintain or improve brain functions. Indeed, a study indicated that MCT supplementation improved cognitive performance in subjects with AD or mild cognitive impairment (MCI) and that cognitive improvement correlated positively with blood levels of beta-hydroxybutyrate [59].

We conducted an eight-month study to investigate whether dietary MCT can increase blood ketone body levels without restricting dietary carbohydrates and proteins and enhance cognitive functions in healthy old dogs [19]. Twenty-four dogs (7.5–11.6 years, 10 males and 14 females) were recruited in this 8-month study. The control diet (CD) was a commercial super premium-type maintenance diet for adult dogs and contained 32% protein, 18.5% fat, 32% carbohydrates, and 2.86% crude fiber. The medium chain triglyceride (MCT) diet was the control diet with 5.5% MCT added. Both diets were isocaloric and contained the same levels of protein, fat, and carbohydrates. Dogs fed the MCT diet had significantly higher blood beta-hydroxybutyrate (BHB) two hours after feeding compared with baseline BHB level or the BHB levels of the control dogs. The resulting blood levels of ketone bodies were safe and much lower than the levels of ketone bodies reported in fasted dogs [101]. These data confirm that dietary MCT can significantly enhance blood ketone bodies without limiting dietary protein and carbohydrates. During the study, the dogs were tested on three cognitive tasks (Landmark, Egocentric, and Oddity). The MCT diet significantly improved spatial learning and memory, and visual-spatial attention in old dogs within one month after feeding. Overall, our study results indicated that the MCT diet significantly improved spatial learning and memory, executive functions, attention, and concept learning in healthy old dogs. These data suggest that in addition to application in people with MCI or AD [59,102], MCT may be a potential solution to enhance cognitive function in healthy senior people.

### 6.2. Nutritional Interventions Targeting Oxidative Damage-Related Brain Aging

Oxidative stress has been proposed as the main cause of aging [103], brain aging, and AD [79,80], and using exogenous antioxidants to reduce oxidative damage has been an active area of anti-aging research. Cotman et al. [104] examined the effects of a test diet containing antioxidants and mitochondrial cofactors on cognitive function in aged dogs. The antioxidants included vitamin E, vitamin C, spinach flakes, tomato pomace, grape pomace, carrot granules, and citrus pulp, and mitochondrial cofactors (alpha-lipoic acid and L-carnitine) were added to the test diet to reduce reactive oxygen species (ROS) production by enhancing the function of aged mitochondria. The test diet significantly improved the ability of aged dogs to learn more difficult tasks compared with the aged dogs fed the control diet [104]. A combination of the above-mentioned test diet [104] and environmental enrichment significantly reduced age-dependent deposition of Aβ1-40 and Aβ1-42 in the temporal cortex and cognitive decline in old dogs at least by increasing brain derived neurotrophic factor (BDNF) in the brains of old dogs [105].

Siwak-Tapp et al. [32] reported a significant loss of neurons (about 30%) in the hilus of the hippocampus in old dogs compared with young dogs. In addition, the authors investigated the effects of the test diet containing antioxidants and mitochondrial cofactors [104] and behavioral enrichment on the numbers of neurons in the hilus of the hippocampus in old dogs in a 2.8-year long feeding study [32]. Interestingly, the test diet failed to reduce the neuron loss in the hilus of the hippocampus compared with the age-matched control dogs while the behavioral enrichment significantly reduced the neuron loss compared with age-matched control dogs. These data indicate that a combination of exogenous antioxidants coming from fruits and vegetables and mitochondrial cofactors may improve cognitive function in old dogs, but were not able to reduce the irreversible neuron loss in old dogs. This is an important lesson for scientists to learn when they try to develop interventions to reduce the irreversible neuron loss in people and pets because an effective anti-aging intervention should be able to not only improve cognitive function but also reduce irreversible neuron loss in people and pets.

In addition to antioxidants, other bioactives including S-adenosyl methionine and apoaequorin were tested in old dogs to enhance cognitive function. Oral S-adenosylmethionine (SAMe) tosylate tablets (Novifit tablets, Virbac) were evaluated in old dogs with signs of cognitive dysfunction in a 2-month clinical study. A 14-item standardized questionnaire was used to evaluate behavior and locomotion difficulties. Compared with the placebo dogs, SAMe-treated dogs showed significant improvement in both activity and awareness at the end of the study. In addition, the aggregate mental impairment score was reduced by more than 50% in 41.2% of the SAMe-treated dogs and 15.8% of placebo dogs [14].

Disturbance of intracellular calcium is associated with aging and may be linked to CDS in dogs [106]. Two 32-day studies were conducted to investigate the effects of apoaequorin, a calcium buffering protein, on cognitive function in aged dogs. In the first study, 23 old beagle dogs were randomized into three cognitively equivalent groups and treated with either a placebo, low (2.5 mg), or high (5 mg) doses of apoaequorin. Cognitive tests were started after three days of treatment and dogs were assessed on discrimination learning, attention, and visuospatial memory tasks. The apoaequorin-treated dogs showed significant improvement in the discrimination learning with the low dose and also the attention tasks with the high dose. In the second study, 24 dogs were randomized into three cognitively equivalent groups and treated with either a 5-mg or 10-mg dose of apoaequorin or with 1 mg/kg selegiline (Anipryl). Dogs were tested on a discrimination learning task and an attention task. The dogs treated with 10-mg apoaequorin showed significantly better performance compared with the dogs treated with selegiline on both tasks. The authors concluded that “these results suggest the calcium-binding protein apoaequorin may have beneficial effects in treating cognitive dysfunction in aged beagle dogs and that apoaequorin is at least as beneficial as selegiline” [106].

### 6.3. Nutritional Interventions Testing Blends of Nutrients and/or Bioactives

Several studies have been done to test blends of nutrients or bioactives in either old dogs or old dogs with CDS. In a 70-day cross-over study, Senilife, containing phosphatidylserine in combination with Gingko biloba extract, pyridoxine, vitamin E and resveratrol, was shown to significantly improve learning and memory in aged dogs [107]. Another study targeted multiple pathological processes including increased inflammation and oxidative damage, and amyloid-beta accumulation in aged dogs [108]. The test blend included turmeric extract, a green tea extract, N-acetyl cysteine, R-alpha lipoic acid, and a black pepper extract. After three months of feeding, dogs fed the test diet had significantly better spatial attention. A clinical study was conducted to determine the effects of Aktivait on the clinical symptoms of CDS in old dogs [109]. Aktivait contained four categories of nutrients including brain strengthening components (DHA/EPA, phosphotidylserine), signaling enhancers (phosphotidylserine, acetyl-L-carnitine, L-carnitine, CoQ10), metabolic enhancers (acetyl-L-carnitine, L-carnitine, CoQ10), and antioxidants (vitamin C, vitamin E, selenium, N-acetyl cysteine, alpha-lipoic acid). Significant improvement was observed in CDS scores for disorientation, changes in interaction, and house soiling behavior at day 21, day 28, and day 42 between the test and the placebo groups [109].

We developed a nutrient blend to test the hypothesis that eliminating or minimizing known risk factors associated with accelerated brain aging and AD in people can retard age-induced decline in cognitive functions in dogs and cats. The first study was undertaken with healthy middle-aged and old cats without any signs of CDS [17] and the second study was conducted on healthy old dogs without any signs of CDS [18]. The nutrients in the blend called the brain protection blend (BPB) were selected based on their ability to eliminate or mitigate known risk factors for brain aging and AD. The BPB blend included antioxidants, arginine, B vitamins, and DHA/EPA to mitigate increased oxidative stress and chronic inflammation, poor vascular healthy and hypertension, high homocysteine, DHA deficiency, and low status of vitamin B6, B 12, and folate [17,18]. Both the control and the BPB diets contained the same levels of protein, fat, and carbohydrates. All the essential nutrients in the control diets met the daily nutrient requirements recommended by the Association of American Feed Control Officials (AAFCO) for adult dogs or cats. All the essential nutrients included in the BPB blends (except for vitamin C, DHA, and EPA, which are not considered essential nutrients for adult dogs or cats) were also added to the control diets in amounts higher than the daily nutrient requirements recommended by AAFCO for adult dogs or cats. However, all the nutrients (antioxidants, arginine, B vitamins, and DHA/EPA) in the BPB diets were higher than those in the control diets.

In the first study, healthy middle-aged and old cats were randomized into two cognitively equivalent groups based on baseline cognitive performance and fed either the control or test diet for 12 months [17]. During the feeding study, cats were tested on several cognitive tasks to evaluate their learning and memory ability and executive functions. The results showed that the cats fed the BPB diet had significantly better performance on egocentric learning task, egocentric reversal task, size discrimination learning task, and delayed non-matching-to-position task compared with the cats fed the control diet.

In the second study, healthy old dogs were randomized into two cognitively equivalent groups based on baseline cognitive performance and fed either the control or test diet for six months [18]. During the feeding study, dogs were tested on several cognitive tasks (two landmark tests, and three egocentric tests) to evaluate their learning and memory ability and executive functions. The results showed that the dogs fed the BPB diet had better performance on land-1 task, and egocentric reversal 1 task and egocentric reversal 2 task than the dogs fed the control diet. Several of the nutrients in the BPB diet including arginine, alpha-tocopherol, DHA, and EPA were significantly higher in the plasma of dogs fed the BPB diet compared with the control dogs. These data indicate that the BPB blends had cognition-enhancing benefits in healthy old dogs and cats without CDS, and dogs and cats needed to consume essential nutrients at levels higher than their daily requirements to protect their brains against aging-induced cognitive decline [17,18].

Since the BPB diet was able to enhance cognitive performance in healthy middle-aged and old cats [17], we conducted a four-year study to determine whether the BPB diet could reduce brain atrophy in middle-aged and old cats [110]. The thickness of the interthalamic adhesion (ITA) significantly declines with aging in both dogs [111] and cats [112], making it a reliable index of brain atrophy. In this study, we investigated the effect of both the BPB diet and the control diet on the changes in ITA thickness in cats. Both the control and test diets were the same diets tested in our previous study [17]. Twenty-four cats (six to nine years of age) were randomized into two groups with 12 cats per group. Cats were fed either a complete and balanced diet or the BPB diet for fifty-three months. A Siemens/Varian 7T magnet scanner was used in the study. A neurologist blind to the dietary assignment manually measured the maximal ITA thickness with VivoQuant v 4.4 software. There was no difference (*p* = 0.8177) in the ITA thickness between the BPB cats (4.93 ± 0.06 mm) and control cats (4.91 ± 0.09 mm) at baseline. At the end of the study, ten cats from each group competed the feeding study, and the % reduction in ITA thickness from baseline was −15.54 ± 1.16% for the control cats (*n* = 10) and −7.95 ± 1.31% for the BPB cats (*n* = 10), indicating that the control cats had significantly (*p* = 0.0004) faster decline in the ITA thickness than the BPB cats. The results confirm that the BPB diet can reduce the atrophy of ITA, indicating that the BPB can slow down brain atrophy in healthy cats. The data from our behavioral and brain imaging studies in the dogs and/or cats indicated that the BPB is able to not only enhance cognitive function bust, also reduce brain atrophy in aged pets, making BPB the optimal nutritional solution to promote healthy brain aging in pets. We believe that our BPB data may also facilitate the development of similar nutritional solutions for healthy brain aging in people.

Recently, we completed a double-blind placebo-controlled clinical study to evaluate the effects of the combination of MCT and the BPB on pet dogs with cognitive dysfunction syndrome (CDS) [20]. Twenty-four participating veterinary clinics screened senior dogs for signs of CDS as determined by a Senior Canine Behavior and Health Questionnaire, which included six categories (disorientation, altered social interaction, sleep–wake cycle disturbance, loss of house training, anxiety and altered activity). Out of 102 dogs screened, 87 were randomly enrolled into one of three diet groups with 29 dogs per group: Control, 6.5% MCT + BPB, 9% MCT + BPB. Each dog’s behavioral signs were re-evaluated at day 30 and day 90. All six categories of the CDS signs were significantly improved (*p* < 0.05, Fisher least significant difference test) in the dogs fed the 6.5% MCT + BPB diet at the end of the 90-day study. In contrast, the control diet did not significantly improve disorientation and social interaction scores. The 9% MCT diet + BPB diet was observed to produce significant improvements in only those dogs whose owners had positive feedback on the diet, suggesting possible palatability issues. These findings support the use of the 6.5% MCT + BPB diet for managing clinical symptoms in senior dogs with CDS.

## 7. Conclusions

**Figure 1:**
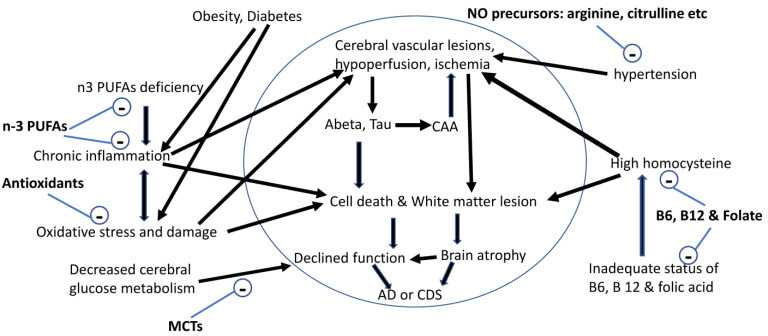
Nutritional management of the risk factors associated with brain aging and AD. Abeta: β-amyloid peptide; AD: Alzheimer’s disease; CAA: cerebral amyloid angiopathy; CDS: cognitive dysfunction syndrome; n-3 PUFAs: omega-3 polyunsaturated fatty acids; NO: nitric oxide; Tau: abnormal hyperphosphorylation of tau.

With improvement in medicine, nutrition, and environment, the lifespans of people and pets have been significantly increased. Longer lifespans increase the risk of many age-related chronic diseases including AD in people and CDS in pets. Both AD and CDS are the consequence of accelerated brain aging and adversely affect the quality of life in people and pets. Brain aging in people and dogs share similar morphological changes including cortical atrophy with irreversible loss of brain cells, cerebral amyloid angiopathy, and ventricular enlargement (Table 1). Currently, both AD and CDS are incurable diseases, therefore, more efforts should be focused on reducing brain atrophy and minimizing the risk of AD in people and CDS in dogs. Many risk factors have been associated with brain aging and AD in people (Figure 1), however, the risk factors associated with brain aging and CDS in dogs have not been fully investigated, but age, gender, oxidative stress, and deficiency of sex hormones appear to be associated with increased risk of accelerated brain aging and CDS in dogs. Since irreversible brain atrophy is the cause of cognitive decline and dementia, an optimal nutritional solution for healthy brain aging must be able to not only enhance cognitive function, but also reduce irreversible brain atrophy. Some studies have investigated the effects of apoaequorin, or S-adenosylmethionine, or blends of nutrients or bioactives such as phosphatidylserine, Gingko biloba extract, pyridoxine, vitamin E, and resveratrol, but only on cognitive function in old dogs. Several studies have been conducted to test the effects of antioxidants from fruits and vegetables and mitochondrial factors on cognitive function and loss of neurons in the hippocampus in old dogs. The results show that the combination of antioxidants and mitochondrial factors enhanced cognitive function, but failed to reduce neuron loss in old dogs. We developed two nutritional solutions (MCT and BPB) that mitigate known risk factors associated with brain aging and AD (Figure 1). Our studies showed that the MCT was able to enhance cognitive function in old dogs, and the BPB blends were able to enhance cognitive function in both dogs and cats. In addition, data from our brain imaging study showed that the BPB can reduce brain atrophy in cats. Another important lesson from our BPB studies in dogs and cats is that consuming RDA levels of essential nutrients is not sufficient for healthy brain aging in dogs and cats, which may also apply to people. A recent clinical study showed that the combination of the MCT and BPB was able to alleviate clinical symptoms of CDS in dogs. Dogs and humans share many similar age-induced changes in the brain (Table 1), which suggests that dogs may be a natural model for understanding brain aging and developing preclinical nutritional solutions in humans. In fact, the BPB was developed based on known risk factors of brain aging and AD in humans (Figure 1), which suggests that humans and dogs share common risk factors associated with brain aging and dementia, and humans are more likely to be able to benefit from the BPB. In addition, MCT can enhance cognitive function in dogs and people, which further supports our hypothesis that targeting known risk factors associated with brain aging and AD can promote healthy brain aging and manage clinical symptoms of dementia in both humans and dogs.

## Figures and Tables

**Table 1:** Age-induced changes in the brains of humans and dogs.

Aging-Related Changes	Human Brains	Dog Brains	Reference
Cortical atrophy	Total brain volumes started to decline in the forties significantly and continued to decline through fifties and seventies. Cortical atrophy is not linear and uniform across brain regions. Increased atrophy rates across all neocortical regions were observed in subjects with clinical signs of cognitive impairment. Medial temporal cortex had greater atrophy rates in subjects with early diseases while greater atrophy rates occurred in prefrontal, parietal, posterior temporal, and cingulate cortex in subjects at later stages of mild cognitive impairment and AD.	Significant decrease in total brain volume was observed only in senior dogs aged 12 years and older. Frontal lobe atrophy developed in the old dogs aged 8–11 years. Hippocampal volume also decreased with age, but occipital lobe did not decline with age. The neuron density was significantly reduced in the brains of dogs with CDS compared with age-matched control dogs.	[1,4,8,21,22,23,24,113]
Cerebral amyloid angiopathy	Amyloid (Aβ1-40) deposits in blood vessel walls.	Amyloid (Aβ1-40) deposits in blood vessel walls.	[1,8]
Ventricular enlargement	Ventricle volume was constant between 20 and 39 years of age, but increased drastically after 40 years of age.	Rapid increase in the ventricle volume was observed in beagle dogs after age of 11.	[3,4,5,23]
Senile Plaques (SP)	The longer, more toxic Aβ1-42 initially accumulated in the brain, followed by the shorter and more soluble Aβ1-40 deposition in SP and blood vessel walls. SPs were widely present in the cortex and hippocampus of AD patients.	Dogs naturally develop SP of the diffuse (non-β-sheet) subtype. Aβ1-42 initially builds up in the brain, followed by Aβ1-40 deposition in diffuse SP and blood vessel walls. In the brains of dogs with CDS, three types of amyloid deposits were detected: diffuse, focal, and vascular deposits.	[1,3,7,8,42]
Neurofibrillary Tangles (NFTs)	NFTs are the consequence of intracellular aggregation of hyperphosphorylated tau protein (p-tau) in neurons and glial cells. NTFs were widespread in the cortex and hippocampus of AD patients.	NFTs have not been detected in dogs. However, p-tau was detected in neurons and astrocytes in dogs with CDS. The number of p-tau-positive cells in the brains were much higher in dogs with CDS than in the normal aged dogs. In addition, the levels of p-tau increased with the ages of dogs with CDS and were significantly associated with the Aβ deposition in dogs with CDS.	[1,3,7,8]
Cerebral glucose metabolism	Cerebral glucose metabolism was reduced in old people, and was further compromised in AD subjects compared with the age-matched controls.	Cerebral glucose metabolism was reduced in middle aged, and further reduced in senior dogs.	[45,47,49,50]
